# Characterization and Comparison of the *CPK* Gene Family in the Apple (*Malus × domestica*) and Other Rosaceae Species and Its Response to *Alternaria alternata* Infection

**DOI:** 10.1371/journal.pone.0155590

**Published:** 2016-05-17

**Authors:** Menghan Wei, Sanhong Wang, Hui Dong, Binhua Cai, Jianmin Tao

**Affiliations:** College of Horticulture, Nanjing Agricultural University, Nanjing, China; Chiba University, JAPAN

## Abstract

As one of the Ca^2+^ sensors, calcium-dependent protein kinase (CPK) plays vital roles in immune and stress signaling, growth and development, and hormone responses, etc. Recently, the whole genome of apple (*Malus × domestica)*, pear (*Pyrus communis*), peach (*Prunus persica*), plum (*Prunus mume*) and strawberry (*Fragaria vesca*) in Rosaceae family has been fully sequenced. However, little is known about the *CPK* gene family in these Rosaceae species. In this study, 123 *CPK* genes were identified from five Rosaceae species, including 37 apple *CPKs*, 37 pear *CPKs*, 17 peach *CPKs*, 16 strawberry *CPKs*, and 16 plum *CPKs*. Based on the phylogenetic tree topology and structural characteristics, we divided the *CPK* gene family into 4 distinct subfamilies: Group I, II, III, and IV. Whole-genome duplication (WGD) or segmental duplication played vital roles in the expansion of the *CPK* in these Rosaceae species. Most of segmental duplication pairs in peach and plum may have arisen from the γ triplication (~140 million years ago [MYA]), while in apple genome, many duplicated genes may have been derived from a recent WGD (30~45 MYA). Purifying selection also played a critical role in the function evolution of *CPK* family genes. Expression of apple *CPK* genes in response to apple pathotype of *Alternaria alternata* was verified by analysis of quantitative real-time RT-PCR (qPCR). Expression data demonstrated that *CPK* genes in apple might have evolved independently in different biological contexts. The analysis of evolution history and expression profile laid a foundation for further examining the function and complexity of the *CPK* gene family in Rosaceae.

## Introduction

Plant growth and crop production are adversely affected by environmental stresses, such as drought, low temperature, high salinity, pathogen infection, microbial elicitors and wounding. To adapt to these environmental conditions, plants have developed a mechanism that includes the perception of stress signals, subsequent signal transduction, and the activation of various physiological and metabolic responses [1]. Calcium ions (Ca^2+^) play a central role as a second messenger in the signal transduction pathways [2]. In plants, the perturbation of cytosolic Ca^2+^ concentration responds to diverse endogenous and external signals, including phytohormone status, abiotic stress and biotic stress, as well as mechanical wounding [3–6]. These calcium signatures can be recognized by different Ca^2+^ sensor molecules which subsequently transduce the signal to downstream signaling cascades such as phosphorylation of target proteins.

In plants, four main classes of Ca^2+^ sensors have been identified, including calmodulin (CaM), calmodulin-like proteins (CML), calcineurin B-like proteins (CBL) and calcium-dependent protein kinases (CPKs) [7–9]. Among them, CPKs are best characterized and constitute a large multigene family, which widely exist in plant kingdom from algae to angiosperms. Structurally, CPK proteins contain four domains, a variable N-terminal domain, a conserved kinase domain, an autoinhibitory region, and a calmodulin-like domain [10]. The highly variable N-terminal domain contains myristoylation or palmotylation sites, which facilitate membrane association [11]. And the calmodulin-like domain contains EF-hands for binding calcium ions through interactions with alpha-helices [12].

CPK proteins have been implicated to participate in immune and stress signaling, growth and development, and hormone responses. Ectopic expression of Arabidopsis *CPK* gene *AtCPK1* enhanced NADPH oxidase activity and oxidative burst[13]. AtCPK1 also activates the accumulation of salicylic acid (SA) [14]. AtCPK5 was reported to activate respiratory burst oxidase homolog D (RBOHD) to induce a reactive oxygen species (ROS) burst [15]. CPKs help to enhance drought tolerance by regulating abscisic acid (ABA) signal transduction (e.g. AtCPK4/11) [16], and via stomatal closure (e.g. AtCPK3/6) [17]. AtCPK23 helps to enhance plant salt tolerance through controlling of K^+^ channels [18]. CPKs also function in hormone signaling, such as ABA [19], MeJA[20] and ethylene[21, 22]. In addition, some *CPK* genes also participate in root development [23] and pollen tube growth [24–26].

Because of the critical regulatory functions of *CPK* genes in plant responses to different stresses and developmental processes, *CPK* gene family have been extensively studied in Arabidopsis (*Arabidopsis thaliana*)[27], rice (*Oryza sativa*) [28], poplar (*Populus trichocarpa*) [29], maize (*Zea mays*) [30], etc. However, compared to the extensive studies in other species, the *CPK* gene family in the Rosaceae has not been widely surveyed. The most economically important fruit, such as apple (*Malus × domestica*), pear (*Pyrus communis*), peach (*Prunus persica*), plum (*Prunus mume*) and strawberry (*Fragaria vesca*), all belong to the Rosaceae family [31]. *M*. *× domestica* is one of the most economically important fruit cultivated worldwide, and 30 *CPK* genes have been identified by bioinformatics [32]. Recently, the whole genome of apple, peach, strawberry, plum, and pear has been fully sequenced. This resource provides an opportunity to further analyze the *CPK* gene family in Rosaceae species. In this study, we aim to analyze the *CPK* genes in apple and other Rosaceae fruit species at whole genome scale, elucidate their evolutionary history, and provide a relatively complete overview of the *CPK* gene family in Rosaceae. Using a HMMER-BLASTP-InterProScan strategy, we identified 37 *CPK* genes from apple, and other 86 *CPK* genes from pear, peach, strawberry and plum. Then, their evolution event were surveyed by phylogenetic and synteny analysis. Furthermore, the expression profiles of *CPK* genes in response to pathogen infection were investigated. The identification and comprehensive study for *CPK* genes from Rosaceae will provide valuable information for breeding biotic stress-resistant fruit tree and further studying of the biological function and evolutionary relationship of this family in Rosaceae.

## Results

### Identification and classification of *CPK* genes in the Rosaceae

A HMMER-BLASTP-InterProScan strategy was used to search for genes encoding CPK proteins. The HMM (Hidden Markov Model) profiles of protein kinase domain (PF00069) and EF-hand domain (PF13499 and PF13202) were downloaded from the Pfam protein family database (http://pfam.sanger.ac.uk/). These HMM profiles were used to search proteomes of apple, pear, peach, plum and strawberry by hmmsearch with the threshold set of the Pfam GA gathering cutoff. Then, the HMMER selected proteins were used for a BLASTP query of the original proteomes. Finally, the BLASTP hits were scanned for kinase and EF-hand domains using InterProScan[33], then truncated sequences and pseudogenes were removed. Initially, 129 nonredundant putative genes were identified. After manually checking the sequences of these genes, 6 genes were removed for having diverse sequences.

In comparison with a previous study[32], in which 30 *MdCPK* genes have been identified, 37 *MdCPK* genes were found in this work, although the apple genome data used is the same version deposited in Phytozome. After carefully checking, we found that the discrepancy in number of *CPK*genes lies in the methods used by these two studies. The previous work used BLASTP to search apple genome using *CPK* genes from *Arabidopsis thaliana*, then all the candidate *CPKs* of apple were scanned using SCAN PROSITE software to confirm the presence of the EF-hands signature motif. We adopted a HMMER-BLASTP-InterProScan strategy that mined the *CPK* genes based on the existence of the HMM profiles of protein kinase domain and EF-hand domain. The advantage of our method is that HMM profile was constructed from some representative *CPKs* from various species, which covered more domain information than that only from Arabidopsis *CPKs*. Our search strategy was tested against Arabidopsis and detected all previously reported 34 *CPKs*, with *AtCPK25* removing for having truncated conserved domain. Using this strategy, we detected 37 *CPK* genes in apple genome, which include all the genes reported by[32] except one gene, MDP0000169895, in which no EF-hand was detected. The existence of protein kinase domain and EF-hand domain were further confirmed by SMART (http://smart.embl-heidelberg.de/), PfAM (http://pfam.xfam.org/), and SUPERFAMILY (http://supfam.org/SUPERFAMILY/). Therefore, additional eight *CPK* genes were identified by this study.

**Fig 1:**
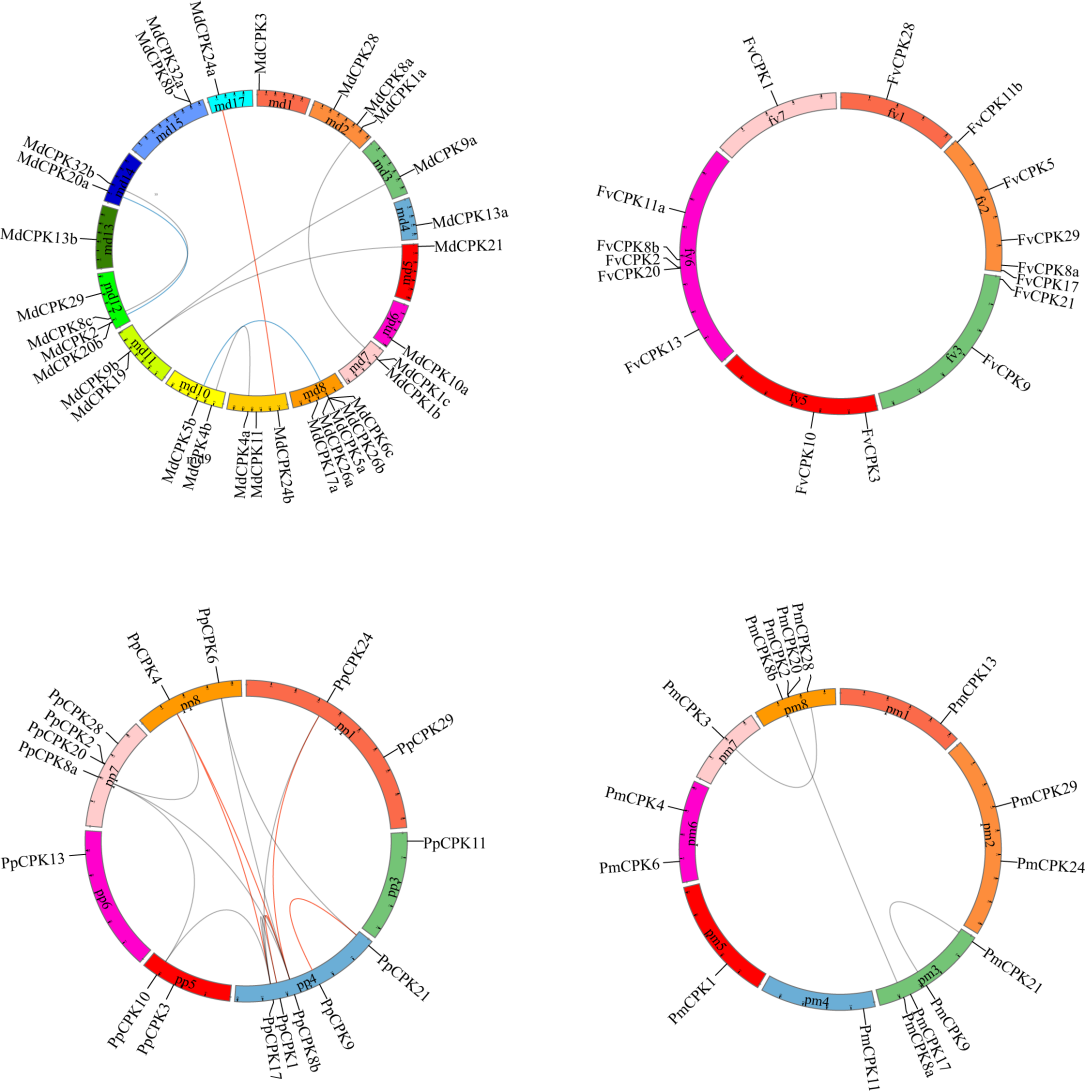
Localization and duplication of the *CPK* genes in the apple, peach, plum and strawberry genome. Circular visualization of the *CPK* genes mapped on the different chromosomes in the genome using the Circos software. Chromosome number is indicated on the chromosome. The synteny relationship between each pair of *CPK* genes were detected by using the MicroSyn software. The genes have synteny relationship are linked by lines. Red link: >30 anchors in a synteny block, blue link: 20–30anchors, green link: 10–20 anchors, gray link: 5–10 anchors.

Finally, a total of 123 *CPK* genes were determined as *CPK* genes in the five species. These *CPK* genes in each species were named based on the similarity with Arabidopsis *CPKs* as the nomenclature in [32]. A total of 37 apple *CPK* (*MdCPK*) genes, 37 pear *CPK* (*PbCPK)* genes, 17 peach CPK (*PpCPK*) genes, 16 strawberry *CPK* (*FvCPK*) genes, and 16 plum *CPK* (*PmCPK*) genes were identified (Table 1, S1–S4 Tables). At the time of analysis, nearly complete genomes were available for apple, peach, strawberry and plum, while pear only had scaffold assemblies data available. In apple genome, chromosome 16 did not contain any *CPK* genes. The highest number of *MdCPK* genes (5, or 13.6% of the total) was found on chromosome 8 (Fig 1). Only one *CPK* gene was located on chromosome 1, 3, 4, 5, 6, and 13, respectively. Similar to that of *MdCPK*s, the distribution of the *CPK* genes in peach, strawberry and plum genomes is random (Fig 1). *CPK* genes in pear can only be anchored to scaffold assemblies now (S1 Table).

**Table 1:** *CPK* genes and related information in apple.

Gene name*	Gene ID	Chr	Start	End	Str	Len	MW	pI
MdCPK1c	MDP0000128057	chr7	5521968	5529057	-	660	73.99	6.40
MdCPK1b	MDP0000142687	chr7	5521790	5528992	-	618	69.01	5.45
MdCPK1a	MDP0000153100	chr2	29570514	29576411	+	566	62.87	4.94
MdCPK2	MDP0000232344	chr12	4101197	4105359	+	775	86.67	6.44
MdCPK20a	MDP0000318339	chr14	5713952	5723780	-	1023	116.65	6.01
MdCPK20b	MDP0000513005	chr12	4295808	4308804	+	679	76.21	5.63
MdCPK5a*	MDP0000162676	chr8	9964949	9969366	-	756	85.71	5.46
MdCPK26b	MDP0000457940	chr8	9964776	9971063	+	1403	158.29	5.59
MdCPK6c*	MDP0000554360	chr8	9981452	9988787	+	581	64.76	6.51
MdCPK5b*	MDP0000306850	chr10	13769805	13772951	+	588	65.74	6.36
MdCPK26a	MDP0000297184	chr8	9975429	9978531	-	571	64.04	6.34
MdCPK4b	MDP0000232885	chr10	5888120	5891732	+	518	58.15	5.74
MdCPK4a	MDP0000260834	chr9	22196543	22200169	-	517	58.17	5.50
MdCPK11	MDP0000494270	chr9	17647795	17649291	+	498	55.59	5.00
MdCPK11a*	MDP0000203275	unanchored	13999639	14000609	-	323	36.74	5.32
MdCPK11c*	MDP0000895339	unanchored	73579661	73580722	-	353	40.10	4.91
MdCPK17b	MDP0000138436	unanchored	20561522	20564258	+	534	59.75	5.92
MdCPK17a	MDP0000802997	chr8	16451427	16454495	+	533	59.69	5.79
MdCPK3*	MDP0000920355	chr1	1097749	1101688	+	501	56.12	6.33
MdCPK29	MDP0000142398	chr12	20910182	20913063	-	527	59.86	6.17
MdCPK21	MDP0000232001	chr5	1385740	1389468	+	554	62.12	6.44
MdCPK9a*	MDP0000320888	chr3	24125216	24129169	-	607	68.35	7.10
MdCPK19	MDP0000180811	chr11	23439921	23443151	-	504	57.10	7.19
MdCPK9b*	MDP0000629000	chr11	23437472	23439940	+	293	33.23	5.67
MdCPK24a	MDP0000262701	chr17	6495752	6498333	+	541	61.70	5.25
MdCPK24b	MDP0000282003	chr9	5964148	5972280	+	954	108.28	5.83
MdCPK8b	MDP0000119457	chr15	40625083	40627959	+	476	52.88	6.39
MdCPK32a	MDP0000649508	chr15	40603145	40610990	-	709	79.03	8.96
MdCPK8a	MDP0000269423	chr2	26382762	26389283	+	553	62.14	7.06
MdCPK8c	MDP0000260857	chr12	6533214	6538860	-	664	75.23	5.86
MdCPK32b	MDP0000179069	chr14	9875913	9880717	-	676	76.66	8.24
MdCPK13a	MDP0000164868	chr4	15515595	15520798	-	585	65.77	6.60
MdCPK13b	MDP0000649496	chr13	15942217	15944688	+	345	39.18	4.97
MdCPK10a	MDP0000218522	chr6	20540708	20544197	+	570	64.33	6.86
MdCPK10b	MDP0000301254	unanchored	24840430	24843853	+	548	62.22	7.32
MdCPK10c	MDP0000308706	unanchored	24860733	24864154	+	548	62.22	7.32
MdCPK28	MDP0000208913	chr2	11285557	11290409	-	626	70.28	9.14

**Note:**Chr: Chromosome; Str: Strand; MW: molecular weight; Len: Amino acid length; pI: Isoelectric point. Gene name in a previous study [32], and

* indicates addition *CPK* genes detected in our study.

### Phylogeny of *CPK* genes

**Fig 2:**
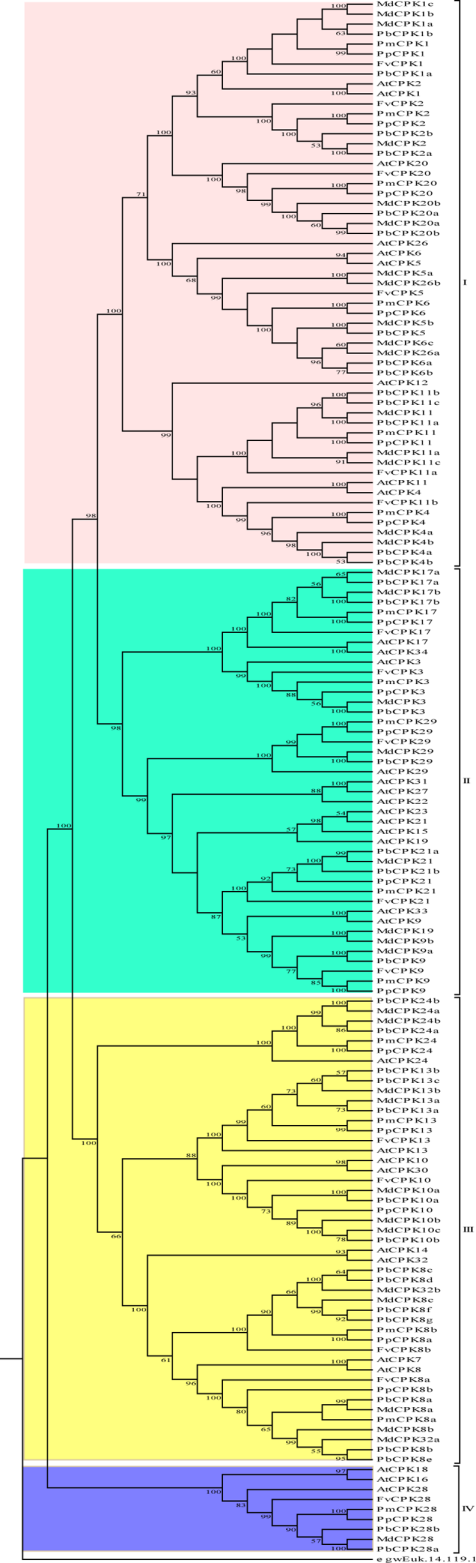
Phylogenetic trees of *CPK* genes in apple, pear, peach, plum, strawberry and Arabidopsis. The phylogenetic tree of *CPK* full length protein sequences was constructed with MEGA6 program with the neighbor-joining method. A *CPK* gene from *Ostreococcus lucimarinus* was used as the outgroup. The numbers beside the branches represent bootstrap values based on 1000 replications.

To detect the evolutionary relationships of *CPK* genes in Rosaceae, we performed phylogenetic analyses on the 123 *CPK* genes from apple, pear, peach, plum and strawberry using Neighbor-Joining (NJ), Minimal Evolution (ME) and Maximum Parsimony (MP) algorithms, respectively. The tree topologies produced by the three methods are largely consistent, with only minor modifications (data not shown). Therefore, the NJ tree was selected for further analysis (Fig 2). Based on the phylogenetic tree topology and previous studies, we divided the *CPK* gene family into 4 distinct groups (subfamilies): Group I, Group II, Group III, and Group IV. Group I contains 16 *CPK* genes from apple, 14 from pear, and 6 from each of strawberry, peach and plum. Group II consists of 8 *MdCPKs*, 7 *PbCPKs*, 5 *FvCPKs*, 5 *PpCPKs* and 5 *PmCPKs*. In Group III, 12 *CPK* genes are from apple, 14 form pear, 4 from strawberry, 5 form peach and 4 from plum. Group IV genes constitute the smallest subfamilies in all of the five species, which contain two *CPK* genes from pear, one from each of other species. The number of *CPK* genes in Group I and Group III from apple and pear were greater than that from strawberry, peach and plum, mainly due to the expansion of *CPK* genes in apple and pear. And the size of Group II in apple and pear were slightly bigger than other three species. These results implied that the variance in the number of *CPK* genes was mainly due to the occurrence of gene gain or loss in subfamilies independently among the different species.

Using a *CPK* gene from *Ostreococcus lucimarinus* as the outgroup for the five Rosaceae species and Arabidopsis *CPKs*, the general topology of the resulting Neighbor–Joining tree appeared similar to that of a previous study [27]: the Group IV lineage appeared to have split first from the last common ancestor. Group III formed a clade close to Groups I and II, while the divergence between Groups I and II occured at the most recent time.

### Gene structure and conserved domains in *MdCPK* genes

**Fig 3:**
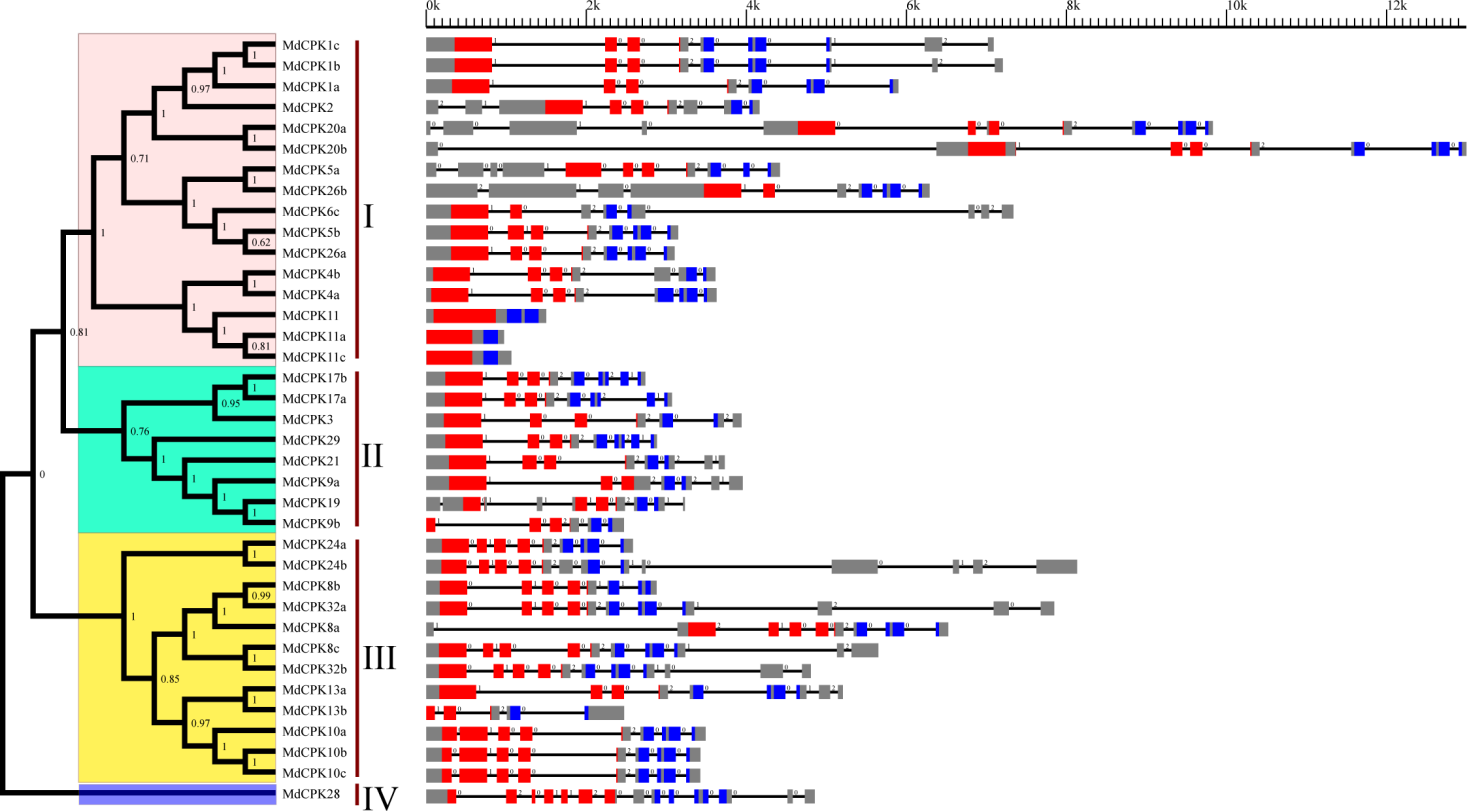
Schematic representations of the exon–intron compositions of *CPK* genes in apple. Phylogenetic tree of Md*CPK* genes are placed on the left. Exons, represented by boxes, were drawn to scale. Dashed lines connecting two exons represent an intron. Protein kinase domain and EF-hand domain is marked in red and blue, respectively.

Most of the apple *CPK* genes have six or seven introns, with clear intron phase patterns (Fig 3). For the members in each subfamily, the intron number and phase pattern on the full length protein sequences are variable, but conserved on the protein kinase domain. Of the 16 members in Group I, 11 genes contain 3 introns with the same phase pattern in kinase domain. Interestingly, three intronless *CPK* genes were found in Group I. These *CPK* genes without introns were also observed in other *Rosaceae* species(pear, peach, plum and strawberry; data not shown), while they have not been reported previously outside *Rosaceae* species, indicating introns may be lost during the evolution of these *CPK* genes in *Rosaceae*. All of the Group II members share the same intron-exon organization as most of the Group I, except *MdCPK9b* and *MdCPK19*. Group III members have 4 introns with the same phase pattern, except *MdCPK13a* and *MdCPK13b*, which contain 3 introns. Compared with the other three groups, Group IV only contains one *MdCPK* gene, which has 6 introns on kinase domain.

**Fig 4:**
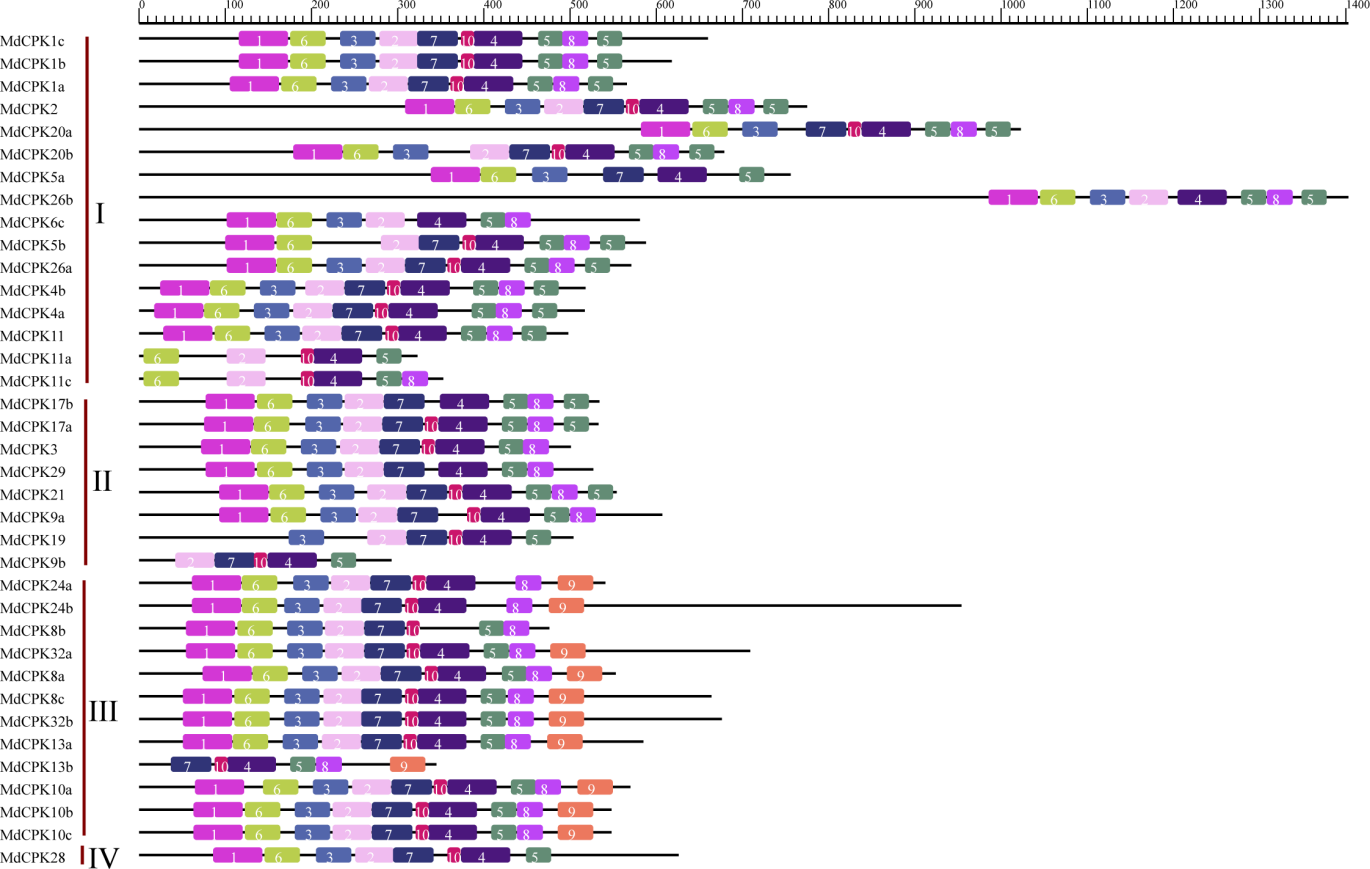
Schematic representations of the conserved motifs of *CPK* genes in apple. Names of genes are indicated on the left. Different motifs are highlighted with different colored boxes with numbers 1 to 10. Lines represent protein regions without detected motif.

The most prominent feature of the proteins in *CPK* gene family is the typical signature domains. We surveyed top 10 motifs in the 37 MdCPK proteins using The Multiple EM for Motif Elicitation (MEME) motif search tool. Protein sequences were also compared with well-characterized signature domains in databases, such as SMART, SUPERFAMILY, Pfam, ProSite and Profiles (data not shown). As showed in Fig 4, motifs 1, 2, 3, 6 and 7 correspond to protein kinase domain; motifs 4, 5, 8 and 9 correspond to calmodulin-like domain; motif 10 corresponds to the junction domain (S5 Table).

### Expansion of the *CPK* gene family in Rosaceae

It is thought that genes in a family usually evolved from multiple gene duplication events. Gene duplication mechanisms mainly include tandem duplication and large segmental/whole-genome duplication (WGD). To examine the relative contribution of these kinds of mechanisms in the expansion of the *CPK* gene family in Rosaceae, we detected the origins of duplicate genes for the *CPK* gene family using the MicroSyn package[34]. Different patterns of gene duplication contributed differentially to the expansion of the *CPK* gene family in the investigated species (Table 2). Remarkably, 15*CPK* genes in apple were duplicated and retained from WGD events, compared to 10 in peach and 6 in plum. No WGD events can be detected between *CPK* genes in strawberry. We did not find tandem duplication events for *CPK* genes in strawberry, peach and plum, however, 10 *CPK* genes in apple genome were found to form 4 tandem duplicated clusters. These results showed that WGD or segmental duplication played vital roles in the expansion of the *CPK* genes in apple, peach and plum. However, for the *CPK* genes in strawberry, other duplication events, such as dispersed gene duplication, is the main source of expansion.

**Table 2:** Duplication genes in apple, peach and plum.

Species	Duplicated pair		Anchors	E value	Mean Ks	SD Ks	Ka/Ks
*Malus × domestica*	MdCPK24b	MdCPK24a	79	1.57E-142	0.28	0.28	0.77
	MdCPK20a	MdCPK20b	11	3.81E-20	0.38	0.36	0.63
	MdCPK26b	MdCPK5b	10	6.41E-16	0.28	0.3	0.56
	MdCPK4a	MdCPK4b	9	2.41E-17	0.26	0.34	0.17
	MdCPK9b	MdCPK9a	8	2.74E-11	0.19	0.1	0.72
	MdCPK32b	MdCPK8c	7	8.04E-07	0.55	0.75	0.54
	MdCPK1b	MdCPK1a	6	6.94E-09	0.13	0.09	0.27
	MdCPK9b	MdCPK21	5	8.78E-05	1.15	0.67	0.26
*Prunuspersica*	PpCPK24	PpCPK8b	41	3.32E-113	0.6	0.67	0.17
	PpCPK8b	PpCPK1	39	3.38E-178	0.79	0.62	0.25
	PpCPK21	PpCPK9	37	3.56E-48	1.29	0.38	0.1
	PpCPK8b	PpCPK4	27	8.44E-24	0.69	0.74	0.16
	PpCPK17	PpCPK4	21	2.54E-18	0.55	0.76	0.14
	PpCPK8b	PpCPK8a	9	5.82E-10	1.07	1.16	0.12
	PpCPK10	PpCPK17	7	2.13E-04	1.14	0.75	0.18
	PpCPK21	PpCPK6	6	5.35E-05	1.32	0.92	0.22
	PpCPK8b	PpCPK17	6	3.48E-04	1.19	0.98	0.15
	PpCPK8a	PpCPK4	6	3.94E-05	1.37	0.47	0.2
	PpCPK8b	PpCPK6	5	4.43E-04	0.95	0.44	0.31
*Prunusmume*	PmCPK21	PmCPK9	7	8.01E-10	1.21	0.37	0.1
	PmCPK8a	PmCPK8b	5	2.09E-05	1.02	0.31	0.13
	PmCPK28	PmCPK3	5	6.22E-04	0.63	0.2	0.74

**Note:** Mean Ks: mean value of each pair of genes within a duplicated block. Ka/Ks: ratio of no-synonymous substitution (Ka) and synonymous substitution (Ks) value of a pair of duplicated genes.

**Fig 5:**
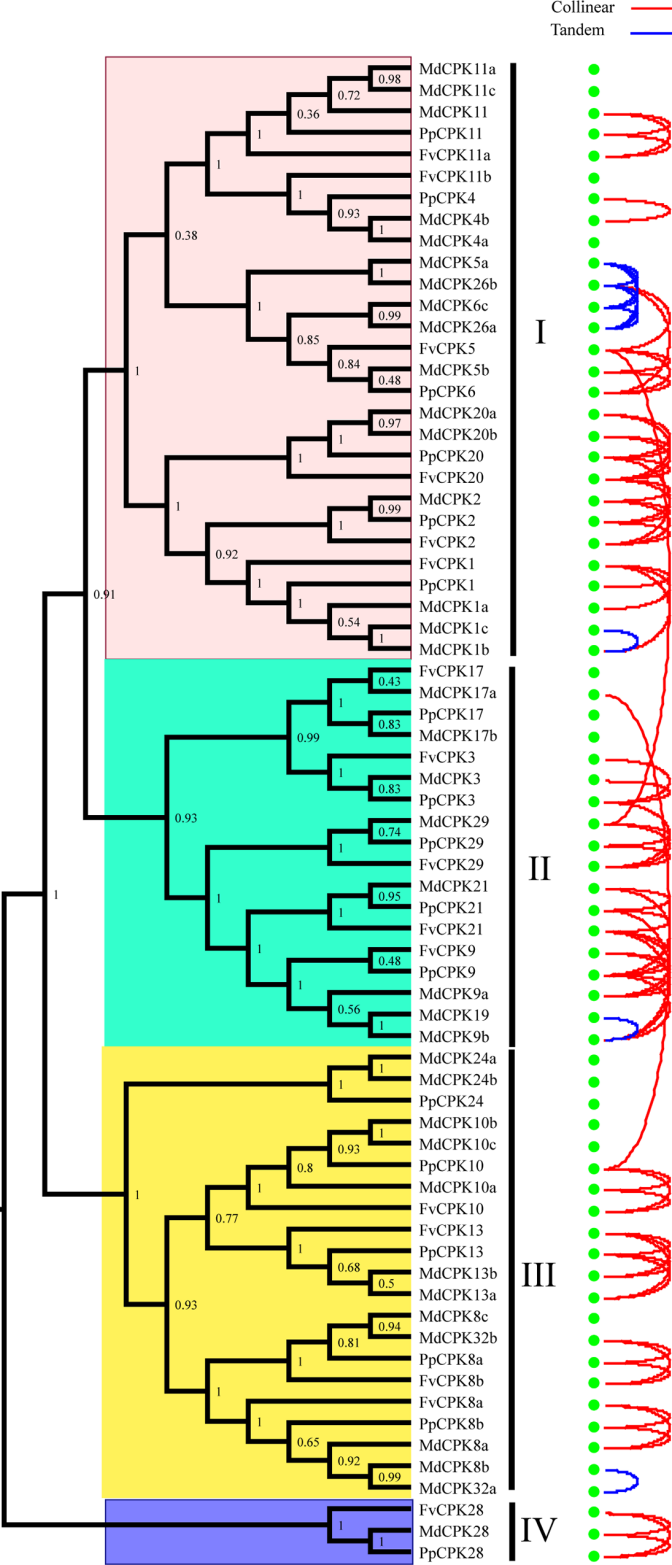
Phylogenetic tree of the *CPK* gene family in apple, peach and strawberry annotated with collinear and tandem relationships. Curves connecting pairs of genes suggest either the collinear relationship (red) or tandem relationship (blue).

With the availability of genome sequence, studies of *CPK* gene family duplication history become possible in part by detecting synteny or clustering and ordering of neighboring matching gene pairs [35]. Using the information of neighboring matching gene pairs between two interested genes, synteny can usually detect duplication event of two chromosome fragments containing interested genes. For the five Rosaceae species investigated here, apple and pear belong to the *Maloideae*, peach and plum belong to the *Prunoideae*, and strawberry belongs to the *Rosoideae*. Owing to the lack of complete genome sequence for pear and computational limit, we select apple from the *Maloideae*, peach from the *Prunoideae* and strawberry from the *Rosoideae* to further survey the origin and evolution of *CPK* genes in Rosaceae. The synteny analysis showed that synteny relationship was mainly detected within subgroups, indicating that if the synteny between two members of a gene family is more significant, these two members evolved from a duplication event more recently (Fig 5, S6 Table). Two syntenies were also detected between subgroups, which indicates that some duplication history can be traced between subgroup. As illustrated in Fig 5, one synteny relationship was detected between subgroup I and II, and subgroup II and III, respectively, which support the relationship of 4 subgroups inferred by the feature of phylogenetic tree [27].

The synteny analysis showed that *CPK* genes can be divided into two types in Rosaceae. The first type of syntenic genes has a single strawberry and plum gene that corresponds to two apple gene, such as *FvCPK1*/*PpCPK1*/*MdCPK1a*/*MdCPK1b*, *FvCPK20*/*PpCPK20*/*MdCPK20a*/*MdCPK20b* (Fig 5). The second type has a single strawberry and peach gene that corresponds to a single apple gene, such as *MdCPK29*/*PpCPK29*/*FvCPK29* and *FvCPK28*/*MdCPK28*/*PpCPK28*. These results provide insights that will assist in understanding of orthologous relationship among *CPK* genes in Rosaceae.

### Ks value and Ka/Ks ratio of *CPK* genes

To estimate the evolutionary dates of the segmental duplication events among the *CPK* gene family, we calculated the synonymous substitution (Ks) values between each pair of duplicated genes. The mean Ks of the duplicated *CPK* gene pairs in the syntenic region are shown in Table 2. The Ks values for the *CPK* gene pairs ranged from 0.13 to 1.15. We found that most of segmental duplication pairs in peach and plum and one pair in apple (*MdCPK13a*/*MdCPK2*; Ks = 1.15) may have arisen from the γ triplication (~140 million years ago [MYA]). While in apple genome, many duplicated gene pairs had lower Ks values (0.19–0.55), suggesting that these duplications may have been derived from the recent WGD (30~45 MYA).

To further detect which selective force has been acted on the evolution of the *CPK* gene family, we also calculated the ratio of non-synonymous to synonymous substitution ratio (Ka/Ks) for 8, 11 and 3 pair of duplicated *CPK* genes in apple, peach and plum, respectively. All the Ka/Ks ratios of gene pairs were less than one, implying that purifying selection was the primary influence on the functional evolution of *CPK* family genes.

### Expression profiles of the *MdCPK* genes in response to *Alternaria alternata*

**Fig 6:**
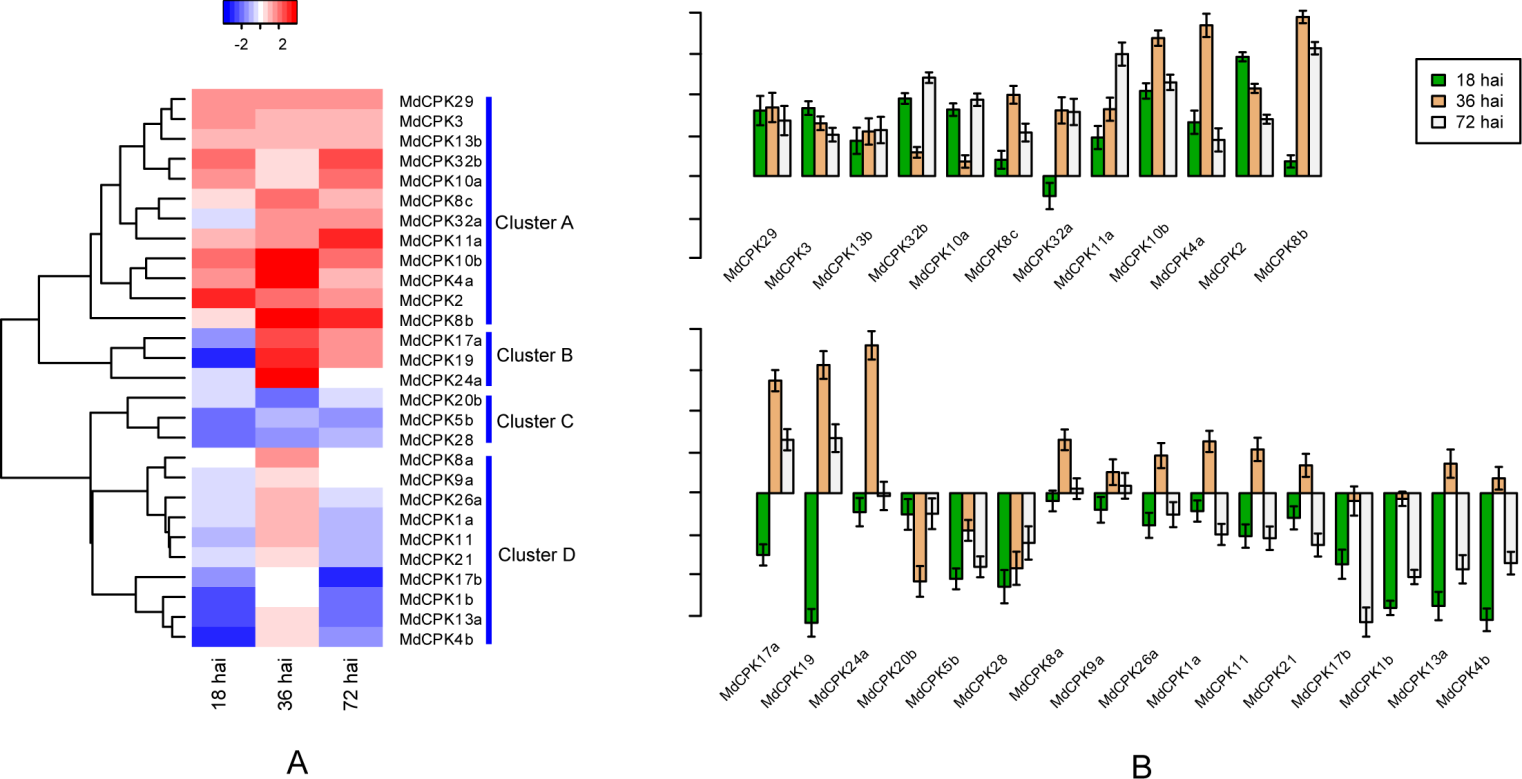
Relative changes in expression of *MdCPK* genes in response to *A*. *alternata* infection using quantitative real-time RT-PCR. The expression of *MdCPK* genes were normalized to tubulin expression. The fold changes of *MdCPK* genes were obtained by calculating the log2 ratio between treated (18, 36 and 72 hours after inoculation (hai) of *A*. *alternata*) and untreated samples. **(A)** The colour scheme in the heat map is blue/red: white indicate a low variation in expression, blue indicate a decrease and red indicate an increase. *MdCPK* gene names are displayed to the right of each row and are clustered in different clusters A, B, C and D using Hierarchical Clustering method. **(B)** Y axis represents the log2 value of fold change of *MdCPK* genes. For example, > 0 means up-regulation and < 0 indicates down-regulation of expression, and “1” represents genes up-regulated with 2 fold change in treated sample relative to control. Bars indicate standard error (SE) in three biological replicates.

Recent studies have provided compelling evidence for the involvement of *CPK*s in most of the immune signaling events. Alternaria blotch disease of apple is one of the most serious fungal diseases, which is caused by the apple pathotype of *Alternaria alternata*. To examine the expression pattern of *MdCPK* genes responding to this pathogen, we examined their expression information using quantitative real-time RT-PCR (qPCR) analysis with RNA from apple leaves sampled at 18 h, 36 h, and 72 h after *A*. *alternata* inoculation (hai). Among the 37 *MdCPK* genes, the qPCR products have been confirmed by sequencing except 9 genes for their unspecific amplification or undetectable expressions, providing a measure of the reliability of the qPCR results of *MdCPK* expression. The sequences of primers were listed in S7 Table. As shown in Fig 6, the expression pattern of *MdCPK* genes in response to *A*. *alternata* can be divided into 4 clusters. Cluster A contains 12 (42.9%) members of detectable genes, which were significantly up-regulated at 18, 36 and 72 hai, except *MdCPK32a*, which showed slight down-regulation at 18 hai. Cluster B contains 3 genes, which exhibited lower abundance of expression at 18 hai, while were highly induced at 36 and 72 hai compared with control. In Cluster C (3 genes), all the *MdCPK* genes were down-regulated after pathogen infection throughout the three time points. Genes in Cluster D are up-regulated at 36 hai, whereas down-regulated at18 and 72 hai, except two genes (*MdCPK1b* and *MdCPK17b*), which displayed no significant changes of expression at 36 hai.

## Discussion

CPKs are encoded by a multigene family, which have been identified and analyzed in many land plant species. The number and composition of *CPK* family members differ in various plants. Ancient polyploidy events (hexaploidization) and additional recent lineage-specific WGDs have presumably resulted in varying numbers of gene family within flowering plants. In this study, the number of the *CPK* gene families identified from the five Rosaceae genomes is diverse. The number of *CPK* genes in apple (37) and pear (37) are greater than that in peach (17), strawberry (16), and plum (16). Apple and pear were suggested to have undergone a recent WGD, while peach, strawberry, and plum did not undergo this event [36]. Therefore, this recent WGD event likely led to the different size of *CPK* genes in these Rosaceae species.

For the five Rosaceae species investigated here, apple and pear belong to the *Maloideae*, peach and plum belong to the *Prunoideae*, and strawberry belongs to the *Rosoideae*. The specification of the *Rosoideae* occurred prior to the split of the *Maloideae* and *Prunoideae*. The phylogenetic analysis showed that *MdCPKs* were closest to *PbCPKs* in the phylogenetic tree, while *PpCPKs* and *PmCPKs* had a closer relationship, which was consistent with the evolutionary history among the three subfamilies.

Different expansion mechanisms, such as genome-wide, tandem, and dispersed duplications, were thought to play a significant role in the expansion of specific gene families in plant genomes[37]. It has been shown that the three whole-genome duplications in Arabidopsis were responsible for more than 90% of the increase in transcription factors, signal transducers, and developmental genes [38]. Recently, genome-wide studies have revealed that the apple and pear genomes have experienced at least two genome duplications, one ancient and one before the apple-pear divergence [39]. In this study, the size of apple and pear *CPK* gene family is as twice as that of peach, strawberry and plum. The results of the synteny analysis verified that the expansion of the *CPK* gene family in apple, peach and plum was mainly derived from WGD or segmental duplications and tandem duplication. In strawberry, only a few or no significant segmental duplications were detected. However, the result of synteny analysis across apple, strawberry and peach revealed that almost all *CPK* genes in strawberry have orthologous genes in apple and peach genome. In strawberry, other duplication events, such as dispersed duplications might be the major drivers for *CPK* gene family expansion. The mean Ks value of the duplicated blocks of *CPK* genes in peach and plum are greater than that of apple, indicating that expansion of *PpCPK* and *PmCPK* genes may have arisen from the γ triplication (~140 MYA). It is worth noticing that the synteny detection algorithm determines the relationship between two members in a gene family by searching for conserved, flanking collinear homologous gene pairs between two genomic fragments. For the ancient duplication events, the original flanking homologous gene pairs were hard to determine, leading to uncertain result of synteny analysis. Therefore, it might be that *CPK* genes in strawberry were evolved from the ancient WGD or segment duplications, but they are difficult to be detected now. Other mechanisms, such as genome rearrangement, gene loss, gene transposition and retrotransposition after the ancient polyploidy event, may also have affected the evolution of the *CPK* gene family in strawberry.

The genes duplicated through WGD might experience three alternative fates during the process of evolution, including (i) one copy may become silenced and lost original functions (nonfunctionalization), (ii) one copy may acquire a novel, beneficial function, while the other copy retained the original function (neofunctionalization) and (iii) both copies may become partition of original functions (subfunctionalization) [40]. It has been shown that the retention of genes duplicated through WGD is biased in plant genomes [41]. In Arabidopsis, genes encoding transcription factors, protein kinases, and ribosomal proteins have been preferentially retained after WGD [42]. It is hypothesized that the overretention of duplicated genes through WGD is strongly correlated with greater structural complexity, highly conserved domains, and lower evolutionary rates in the plant genome. Multiple models may simultaneously drive the evolution of genes duplicated after WGDs. In this study, the *CPK* gene family has undergone specific expansion and been preferentially retained in Rosaceae. Rosaceae *CPK* family genes contain several highly conserved functional domains, and present lower Ka/Ks ratios, corresponding to a slower evolutionary rate. These results implied that functions of the duplicated *CPK* genes in Rosaceae did not diverge much during subsequent evolution. These stable function of *CPK* genes over recent years may serve as good targets for dosage balance selection[43].

To resist pathogen, plants have evolved two defense mechanisms to sense pathogens invasion [44]. On the cell-surface of host, pathogen-associated molecular patterns (PAMPs) are recognized by pattern recognition receptors (PRRs), which are subsequently stimulated to trigger PAMP-triggered immunity (PTI).In response, pathogens have evolved the means to suppress PTI by secreting effectors inside the plant cell. These pathogen effectors are recognized by intracellular nucleotide-binding leucine-rich repeat (NB-LRR) immune sensors, which activate the second type of immune defense mechanism, effector-triggered immunity (ETI). PAMPs initiate an influx of calcium ions and an oxidative burst, followed by activation of MAPK and calcium-dependent protein kinase [45, 46]. In these studies, the expression pattern of *MdCPK* genes after *A*. *alternata* infection was surveyed by qPCR. These genes included 10 *MdCPKs* from Group I, 8 from Group II, 9 from Group III and 1 from Group IV. In Arabidopsis, the expression of *AtCPK1* from Group I is rapidly induced by fungal elicitors. Over-expression of *AtCPK1* confers broad-spectrum resistance to bacteria and fungi. Long-term *AtCPK1* over-expression triggered salicylic acid (SA) accumulation and constitutive expression of SA-regulated defense and resistance genes [14]. *MdCPK2*, a closest homolog of *AtCPK1*, displayed continuously up-regulation during the three time points (18, 36 and 72 hai). A functional genomic screen identified four related *AtCPKs* in Group I (*AtCPK4*, *AtCPK5*, *AtCPK6* and *AtCPK11*), as early transcriptional regulators in MAMP signaling [47]. The closest homolog of *AtCPK4* and *AtCPK11* are *MdCPK4b* and *MdCPK4a*. The transcript accumulation of *MdCPK4b* reduced at early stage of infection, and subsequently increased 36 hai, and then declined 72 hai, whereas *MdCPK4a* showed continuously up-regulation. These results indicate that some *CPKs* from Group I exhibittransient and sustained transcriptional modifications upon pathogen infection. Interestingly, in this study, most of the members (77.8%) in Group III exhibited continuously up-regulation at the three time points, suggesting their conserved function in plant defense response. By contrast, several *MdCPKs* (*MdCPK17b*, *MdCPK1b*, *MdCPK28*, *MdCPK5b*, and *MdCPK20b*) showed down-regulation throughout all the treatments. These results suggest that *CPK* genes in apple might have evolved independently in different biological contexts.

## Conclusions

A total of 123 *CPK* genes were identified in the five Rosaceae genomes. Based on the phylogenetic tree topology and structural characteristics, these *CPK* genes were divided into 4 distinct subfamilies (Group I, II, III and IV). Collinearity analysis showed that many duplicated genes in apple genome may have been evolved from a recent WGD (30~45 MYA), whereas, most of segmental duplication genes in peach and plum may have arisen from the γ triplication (~140 MYA). Purifying selection is the major force driving the function evolution of *CPK* family genes. qPCR evidence showed that *MdCPKs* genes might have evolved independently in different biological contexts. These results in this study laid a foundation for further examining the function and complexity of the *CPK* gene family in the Rosaceae.

## Methods

### Gene Identification

The genome sequences of apple, peach, and strawberry were downloaded from Phytozome (http://phytozome.jgi.doe.gov/pz/portal.html#). The pear genome sequence was downloaded from the pear genome project(http://peargenome.njau.edu.cn/), and the plum genome sequence was downloaded from the *Prunus mume* Genome Project (http://prunusmumegenome.bjfu.edu.cn/index.jsp).The complete genome, proteome sequences of Arabidopsis was obtained from The Arabidopsis Information Resource (version 10; http://www.arabidopsis.org). In proteome datasets, if two or more protein sequences at the same locus were identical where they overlapped, we selected the longest sequence. The HMM profiles of protein kinase domain Pkinase (PF00069) and EF-hand domain (PF13499 and PF13202) were downloaded from the Pfam protein family database (http://pfam.sanger.ac.uk/). HMMER was used to search a customized database containing the proteome with the threshold set of the Pfam GA gathering cutoff. The HMMER selected proteins were used for a BLASTP query of the original protein database. Finally, the BLASTP hits were scanned for protein kinase domain Pkinase and EF-hand domains using InterProScan.

### Phylogenetic tree building and protein motif prediction

The amino acid sequences of full length sequences were aligned using the MUSCLE [48] with default parameters. Phylogenetic trees for the aligned sequences were constructed using NJ, ME and MP methods. The statistical support of the retrieved topology was assessed using a bootstrap analysis with 1000 replicates for trees. The conserved motifs in the proteins were detected by MEME (http://meme.nbcr.net/meme/cgibin/meme.cgi), with the following parameters: number of repetitions: any; maximum number of motifs: 20; the optimum motif widths: 6–200 amino acid residues.

### Chromosomal distribution and gene duplication

The genes were plotted separately onto the chromosomes according to their locations on the chromosomes in the GFF file. Genes with a maximum of 5 genes distance were considered to be tandem duplicates. The syntenies between each pair of members were detected by using the MicroSyn software. The parameters were set as follows: window size of 100 genes, tandem gap value of 2, expected threshold value cut off of 1e-10, and 8 homologous pairs to define a syntenic segment. The mean Ks values of orthologous gene pairs in the same synteny block, the Ka and Ks were calculated by Microsyn.

### Plant materials and pathogen treatments

To examine the expression of *MdCPK* genes, the leaves of cultivar, ‘Red Delicious’, inoculated with *A*.*alternata* were used. *A*. *alternata* was expanded on potato dextrose agar (PDA; 200 g potato extract, 20 g dextrose, 20 g agar, 1L water) medium for 5 d at 26°C under dark conditions. The inoculation method was carried out according to the protocol described previously[49]. The mycelia were punched using a hole puncher (DIA. = 5 mm). Six pieces of mycelium ‘cake’ were put on each side of midrib of the abaxial leaf surface, and then incubated at 25°C under a 14 h light/10 h dark cycle in sterilized plastic chambers. Leaves were sampled at 0, 18, 36, and 72 hours after inoculation.

### Gene expression analysis by quantitative real-time PCR

Quantitative real-time PCR was carried out on three independent biological replicates of each sample, as well as three technical replicates for each reaction. Total RNA was extracted using the CTAB method. The qualities and quantities of RNA were determined by 1.5% agarose gel electrophoresis and Nanodrop ND-1000 Spectrophotometer (Thermo Fisher Scientific Inc.; USA), respectively. A SMART™ PCR cDNA Synthesis kit was used to synthesize the cDNA from 1μg total RNA. Primers of *MdCDPK* genes for qPCR were designed using Beacon Designer v7.0 (Premier Biosoft International, California, USA). The real-time quantification was performed using an ABI7300 Detection System using SYBR Green qRT PCR kits (TaKaRa, Toyoto, Japan). PCR mixtures (final volume, 20 μL) contained 10 μL of 2×SYBR Premix, 2 μL of cDNA template, 200 nM of each primer. The PCR reaction was performed as the following conditions: for 3 min denaturation at 94°C followed by 40 cycles of 94°C for 20 s, 60°C for 20 s, and 72°C for 40 s. Following amplification, melting curves were determined with the following program: 95°C for 15 s, 60°C for 1 min, and 95°C for 15 s. For the accuracy of results, each reaction was conducted in triplicate. Expression levels of the selected genes were normalized to tubulin expression. 2^-ΔΔCt^ method [ΔΔCt = (Ct_target gene_−Ct_tubulin gene_) _treatment_−(Ct_target gene_–Ct_tubulin gene_) _control_] was used to calculate the relative expression of *MdCDPK* genes. The gene expression levels were visualized as the heat map and histogram based on the value of log_2_(2^-ΔΔCt^). Heatmaps were generated using R package ‘gplots’ (http://www.bioconductor.org/). Clustering in the heatmap was carried out using Hierarchical Clustering by hclust function in R (http://www.r-project.org/).

## Supporting Information

S1 Table *CPK* genes and related information in pear. (DOC)

S2 Table *CPK* genes and related information in strawberry. (DOC)

S3 Table *CPK* genes and related information in peach. (DOC)

S4 Table *CPK* genes and related information in plum. (DOC)

S5 Table Consensus sequences of motifs in *CPK* genes in apple detected by MEME. (DOC)

S6 Table Synteny relationship between apple, strawberry and peach. (DOC)

S7 Table Primer sequences used for real-time PCR analysis. (XLS)
