# Supplementary material for: Characterization and Comparison of the CPK Gene Family in the Apple (Malus × domestica) and Other Rosaceae Species and Its Response to Alternaria alternata Infection
Source: PLoS One. 2016 May 17;11(5):e0155590. doi: 10.1371/journal.pone.0155590 (PMC4871508; doi:10.1371/journal.pone.0155590)
Supplement: S1 Table — (DOC) [file pone.0155590.s001.doc]

**S1 Table. *CPK* genes and related information in pear**

| **Gene name** | **Gene ID** | **Group** | **Scaffold** | **Start** | **End** | **Str** | **Len** | **MW** | **pI** |
| --- | --- | --- | --- | --- | --- | --- | --- | --- | --- |
| PbCPK1a | Pbr028710 | I | scaffold48.0.1 | 267805 | 276819 | + | 580 | 65.23 | 4.49 |
| PbCPK1b | Pbr033365 | I | scaffold609.0.1 | 1574 | 11084 | + | 739 | 82.64 | 4.73 |
| PbCPK2a | Pbr001322 | I | scaffold103.0 | 197791 | 201038 | - | 657 | 73.64 | 6.11 |
| PbCPK2b | Pbr010307 | I | scaffold17.0.2 | 269684 | 272717 | - | 647 | 71.62 | 6.19 |
| PbCPK20a | Pbr001308 | I | scaffold103.0 | 43533 | 49598 | + | 548 | 61.02 | 6.46 |
| PbCPK20b | Pbr010295 | I | scaffold17.0.2 | 138152 | 143718 | + | 604 | 67.23 | 6.10 |
| PbCPK5 | Pbr032128 | I | scaffold575.0 | 60819 | 66319 | - | 570 | 63.74 | 6.18 |
| PbCPK6a | Pbr023960 | I | scaffold372.0 | 73519 | 78114 | - | 571 | 63.92 | 6.34 |
| PbCPK6b | Pbr033297 | I | scaffold605.0 | 229964 | 234140 | + | 571 | 63.92 | 6.34 |
| PbCPK4a | Pbr018253 | I | scaffold271.0 | 364036 | 367609 | + | 521 | 58.59 | 5.73 |
| PbCPK4b | Pbr040137 | I | scaffold874.0 | 34452 | 38021 | - | 521 | 58.59 | 5.73 |
| PbCPK11a | Pbr027545 | I | scaffold452.0 | 268654 | 270909 | - | 498 | 55.63 | 4.86 |
| PbCPK11b | Pbr033411 | I | scaffold61.0 | 573141 | 574649 | + | 502 | 55.93 | 4.91 |
| PbCPK11c | Pbr033416 | I | scaffold61.0 | 691545 | 693053 | - | 502 | 55.93 | 4.91 |
| PbCPK17a | Pbr018323 | II | scaffold274.0.1 | 41537 | 43579 | + | 338 | 38.53 | 4.51 |
| PbCPK17b | Pbr029596 | II | scaffold5.0 | 1844773 | 1847849 | + | 534 | 59.75 | 5.92 |
| PbCPK3 | Pbr024654 | II | scaffold389.0 | 238390 | 243297 | + | 525 | 58.94 | 6.51 |
| PbCPK29 | Pbr005793 | II | scaffold13.0.1 | 270121 | 276518 | - | 1144 | 127.09 | 7.29 |
| PbCPK9 | Pbr039714 | II | scaffold86.0 | 64426 | 68155 | + | 544 | 61.06 | 6.78 |
| PbCPK21a | Pbr000148 | II | scaffold1.0.1 | 923531 | 927355 | - | 543 | 60.80 | 6.77 |
| PbCPK21b | Pbr031892 | II | scaffold57.0 | 562458 | 565758 | + | 546 | 61.05 | 6.51 |
| PbCPK28a | Pbr023342 | IV | scaffold357.0 | 433423 | 438134 | - | 556 | 63.26 | 9.64 |
| PbCPK28b | Pbr034839 | IV | scaffold66.0.1 | 490045 | 494053 | - | 552 | 62.19 | 9.46 |
| PbCPK13b | Pbr028878 | III | scaffold480.0.1 | 216158 | 220679 | + | 527 | 59.34 | 6.40 |
| PbCPK13c | Pbr028879 | III | scaffold480.0.1 | 300031 | 304552 | - | 527 | 59.34 | 6.40 |
| PbCPK13a | Pbr021635 | III | scaffold322.0 | 158839 | 164934 | + | 527 | 59.44 | 6.29 |
| PbCPK10a | Pbr011500 | III | scaffold180.0 | 473038 | 476965 | - | 549 | 61.97 | 6.96 |
| PbCPK10b | Pbr036114 | III | scaffold708.0 | 118024 | 121988 | - | 548 | 62.31 | 7.15 |
| PbCPK8b | Pbr011659 | III | scaffold182.0.1 | 522096 | 525361 | - | 533 | 59.62 | 6.25 |
| PbCPK8e | Pbr017213 | III | scaffold257.0 | 459785 | 463533 | - | 533 | 59.62 | 6.25 |
| PbCPK8a | Pbr006943 | III | scaffold138.0.1 | 577377 | 581291 | - | 533 | 59.77 | 6.25 |
| PbCPK8c | Pbr017027 | III | scaffold251.0 | 86363 | 89475 | - | 548 | 61.87 | 6.46 |
| PbCPK8d | Pbr017041 | III | scaffold251.0 | 473392 | 476496 | + | 517 | 58.26 | 6.40 |
| PbCPK8f | Pbr023408 | III | scaffold359.0 | 238798 | 242996 | - | 531 | 59.83 | 5.91 |
| PbCPK8g | Pbr037537 | III | scaffold766.0 | 147207 | 176873 | - | 754 | 84.92 | 6.87 |
| PbCPK24b | Pbr011310 | III | scaffold18.0.1 | 544292 | 546857 | - | 358 | 40.09 | 4.87 |
| PbCPK24a | Pbr030700 | III | scaffold53.0.1 | 1067443 | 1069633 | + | 306 | 34.91 | 4.36 |

**Note:** Str: Strand; MW: molecular weight; Len: Amino acid length; pI: Isoelectric point.
