# Supplementary material for: Characterization and Comparison of the CPK Gene Family in the Apple (Malus × domestica) and Other Rosaceae Species and Its Response to Alternaria alternata Infection
Source: PLoS One. 2016 May 17;11(5):e0155590. doi: 10.1371/journal.pone.0155590 (PMC4871508; doi:10.1371/journal.pone.0155590)
Supplement: S2 Table — (DOC) [file pone.0155590.s002.doc]

**S2 Table. *CPK*** genes and related information in strawberry

| **Gene name** | **Gene ID** | **Group** | **Chr** | **Start** | **End** | **Str** | **Len** | **MW** | **pI** |
| --- | --- | --- | --- | --- | --- | --- | --- | --- | --- |
| FvCPK2 | gene18254-v1.0-hybrid | I | LG6 | 18045101 | 18049749 | - | 708 | 78.88 | 6.26 |
| FvCPK1 | gene23668-v1.0-hybrid | I | LG7 | 12250784 | 12264777 | + | 1083 | 114.64 | 5.05 |
| FvCPK20 | gene18135-v1.0-hybrid | I | LG6 | 17846926 | 17855906 | + | 709 | 78.79 | 6.06 |
| FvCPK5 | gene17341-v1.0-hybrid | I | LG2 | 10179942 | 10183296 | + | 568 | 63.59 | 6.07 |
| FvCPK11a | gene05409-v1.0-hybrid | I | LG6 | 27459291 | 27460787 | - | 498 | 55.94 | 4.70 |
| FvCPK11b | gene27440-v1.0-hybrid | I | LG2 | 429217 | 432145 | + | 490 | 55.35 | 5.50 |
| FvCPK17 | gene15357-v1.0-hybrid | II | LG2 | 24311895 | 24319062 | + | 680 | 75.31 | 7.01 |
| FvCPK3 | gene31992-v1.0-hybrid | II | LG5 | 2203768 | 2208038 | - | 548 | 61.27 | 5.75 |
| FvCPK29 | gene08576-v1.0-hybrid | II | LG2 | 19182892 | 19186797 | + | 556 | 62.85 | 6.11 |
| FvCPK9 | gene03391-v1.0-hybrid | II | LG3 | 13177898 | 13182279 | + | 541 | 61.09 | 6.99 |
| FvCPK21 | gene19615-v1.0-hybrid | II | LG3 | 440791 | 444005 | + | 543 | 61.07 | 6.56 |
| FvCPK10 | gene09567-v1.0-hybrid | III | LG5 | 11050829 | 11058423 | + | 960 | 107.88 | 9.10 |
| FvCPK13 | gene13451-v1.0-hybrid | III | LG6 | 6842534 | 6846941 | - | 527 | 59.23 | 6.46 |
| FvCPK8a | gene14687-v1.0-hybrid | III | LG2 | 23420992 | 23425402 | + | 521 | 58.28 | 5.81 |
| FvCPK8b | gene25220-v1.0-hybrid | III | LG6 | 19324137 | 19327350 | - | 549 | 62.08 | 6.67 |
| FvCPK28 | gene14609-v1.0-hybrid | IV | LG1 | 7913710 | 7917475 | - | 550 | 62.20 | 9.13 |

**Note:** Chr: Chromosome; Str: Strand; MW: molecular weight; Len: Amino acid length; pI: Isoelectric point.
