# Supplementary material for: Characterization and Comparison of the CPK Gene Family in the Apple (Malus × domestica) and Other Rosaceae Species and Its Response to Alternaria alternata Infection
Source: PLoS One. 2016 May 17;11(5):e0155590. doi: 10.1371/journal.pone.0155590 (PMC4871508; doi:10.1371/journal.pone.0155590)
Supplement: S3 Table — (DOC) [file pone.0155590.s003.doc]

**S3 Table. *CPK* genes and related information in peach**

| **Gene name** | **Gene ID** | **Group** | **Chr** | **Start** | **End** | **Str** | **Len** | **MW** | **pI** |
| --- | --- | --- | --- | --- | --- | --- | --- | --- | --- |
| PpCPK2 | ppa002734m.g | I | scaffold_7 | 12823736 | 12826713 | - | 639 | 71.30 | 6.24 |
| PpCPK1 | ppa003542m.g | I | scaffold_4 | 21577698 | 21584701 | + | 567 | 63.19 | 5.19 |
| PpCPK20 | ppa003092m.g | I | scaffold_7 | 12724445 | 12729905 | - | 605 | 67.28 | 5.14 |
| PpCPK6 | ppa003459m.g | I | scaffold_8 | 17381330 | 17385040 | + | 573 | 64.30 | 6.39 |
| PpCPK11 | ppa004580m.g | I | scaffold_3 | 1711490 | 1713101 | + | 502 | 56.53 | 4.97 |
| PpCPK4 | ppa004665m.g | I | scaffold_8 | 7108411 | 7113030 | + | 497 | 55.73 | 4.93 |
| PpCPK17 | ppa026653m.g | II | scaffold_4 | 22960834 | 22963576 | - | 534 | 59.76 | 5.79 |
| PpCPK3 | ppa004162m.g | II | scaffold_5 | 11503594 | 11508680 | - | 526 | 59.01 | 6.46 |
| PpCPK29 | ppa004027m.g | II | scaffold_1 | 32253459 | 32256660 | - | 534 | 60.38 | 6.33 |
| PpCPK9 | ppa003830m.g | II | scaffold_4 | 13342829 | 13347541 | + | 545 | 61.17 | 6.90 |
| PpCPK21 | ppa006748m.g | II | scaffold_4 | 1002107 | 1005710 | - | 397 | 44.87 | 4.92 |
| PpCPK24 | ppa021005m.g | III | scaffold_1 | 16622683 | 16625601 | + | 526 | 59.68 | 5.76 |
| PpCPK13 | ppa004141m.g | III | scaffold_6 | 24253001 | 24258038 | - | 527 | 59.44 | 6.29 |
| PpCPK10 | ppa003795m.g | III | scaffold_5 | 15354135 | 15358116 | + | 548 | 62.20 | 7.30 |
| PpCPK8a | ppa004069m.g | III | scaffold_7 | 9889985 | 9894870 | - | 531 | 60.21 | 6.78 |
| PpCPK8b | ppa006164m.g | III | scaffold_4 | 18649008 | 18652908 | + | 425 | 48.05 | 5.49 |
| PpCPK28 | ppa003676m.g | IV | scaffold_7 | 17542990 | 17547513 | + | 557 | 62.97 | 9.79 |

**Note:** Chr: Chromosome; Str: Strand; MW: molecular weight; Len: Amino acid length; pI: Isoelectric point.
