# Supplementary material for: Characterization and Comparison of the CPK Gene Family in the Apple (Malus × domestica) and Other Rosaceae Species and Its Response to Alternaria alternata Infection
Source: PLoS One. 2016 May 17;11(5):e0155590. doi: 10.1371/journal.pone.0155590 (PMC4871508; doi:10.1371/journal.pone.0155590)
Supplement: S4 Table — (DOC) [file pone.0155590.s004.doc]

**S4 Table. *CPK* genes and related information in plum**

| **Gene name** | **Gene ID** | **Group** | **Chr** | **Start** | **End** | **Str** | **Len** | **MW** | **pI** |
| --- | --- | --- | --- | --- | --- | --- | --- | --- | --- |
| PmCPK1 | Pm017091 | I | Pm5 | 7971082 | 7977817 | - | 539 | 60.19 | 5.04 |
| PmCPK2 | Pm026064 | I | Pm8 | 7481694 | 7484694 | + | 630 | 70.38 | 6.32 |
| PmCPK20 | Pm026079 | I | Pm8 | 7569223 | 7574647 | + | 619 | 68.99 | 5.46 |
| PmCPK6 | Pm020686 | I | Pm6 | 4404203 | 4407462 | + | 573 | 64.24 | 6.26 |
| PmCPK11 | Pm013203 | I | Pm4 | 2459927 | 2461435 | + | 502 | 56.51 | 4.92 |
| PmCPK4 | Pm022189 | I | Pm6 | 14941879 | 14946305 | - | 497 | 55.78 | 4.99 |
| PmCPK17 | Pm012231 | II | Pm3 | 17715387 | 17718099 | - | 534 | 59.76 | 6.04 |
| PmCPK3 | Pm023987 | II | Pm7 | 9736742 | 9747850 | + | 1113 | 124.46 | 8.40 |
| PmCPK29 | Pm006154 | II | Pm2 | 15149171 | 15152466 | - | 534 | 60.41 | 6.45 |
| PmCPK21 | Pm009790 | II | Pm3 | 940220 | 943249 | + | 397 | 44.86 | 4.92 |
| PmCPK9 | Pm011831 | II | Pm3 | 14012305 | 14016582 | - | 545 | 61.19 | 6.81 |
| PmCPK24 | Pm007589 | III | Pm2 | 26332496 | 26337814 | + | 596 | 67.49 | 5.53 |
| PmCPK13 | Pm002913 | III | Pm1 | 22164004 | 22168153 | + | 527 | 59.44 | 6.29 |
| PmCPK8a | Pm012379 | III | Pm3 | 19277893 | 19281650 | - | 533 | 59.93 | 6.29 |
| PmCPK8b | Pm025893 | III | Pm8 | 6191749 | 6195770 | + | 438 | 49.38 | 6.78 |
| PmCPK28 | Pm026757 | IV | Pm8 | 11588959 | 11592896 | + | 557 | 62.91 | 9.62 |

**Note:** Chr: Chromosome; Str: Strand; MW: molecular weight; Len: Amino acid length; pI: Isoelectric point.
