# Supplementary material for: Characterization and Comparison of the CPK Gene Family in the Apple (Malus × domestica) and Other Rosaceae Species and Its Response to Alternaria alternata Infection
Source: PLoS One. 2016 May 17;11(5):e0155590. doi: 10.1371/journal.pone.0155590 (PMC4871508; doi:10.1371/journal.pone.0155590)
Supplement: S5 Table — (DOC) [file pone.0155590.s005.doc]

**S5 Table****. Consensus sequences of motifs in *CPK* genes in apple detected by MEME**

| **Motif ID** | **Motif consensus sequences** |
| --- | --- |
| Motif 1 | DLYSLGRELGRGQFGITYLCTEKATGExYACKSISKRKLITAEDVEDVRREIQIMHH |
| Motif 2 | DIVGSPYYVAPEVLRRNYGPEVDVWSAGVILYILLSGVPPFWAET |
| Motif 3 | CHSLGVMHRDLKPENFLFVNKDEDAPLKAIDFGLSVFFKPG |
| Motif 4 | DSAVLSRLKQFSAMNKLKKMALRVIAENLSEEEIAGLKEMFKMMDTDNSGTITFEEL |
| Motif 5 | DLMEAADVDNDGTIDYGEFVAATLHLNKL |
| Motif 6 | GHPNIVTIKGAYEDAVAVHLVMELCAGGELFDRIIARGHYS |
| Motif 7 | EQGIFDAILRGVIDFESDPWPSISDSAKDLVRKMLEPDPKKRLTAHE |
| Motif 8 | EREEHLHAAFSYFDKDGSGYITIDELRQAC |
| Motif 9 | VDTDKDGRISYEEFVAMMKTGTDWRKASRQYSRERFNSLSL |
| Motif 10 | HPWIQNDGKAPDKPL |
