# Supplementary material for: Characterization and Comparison of the CPK Gene Family in the Apple (Malus × domestica) and Other Rosaceae Species and Its Response to Alternaria alternata Infection
Source: PLoS One. 2016 May 17;11(5):e0155590. doi: 10.1371/journal.pone.0155590 (PMC4871508; doi:10.1371/journal.pone.0155590)
Supplement: S6 Table — (DOC) [file pone.0155590.s006.doc]

**S6 Table. Synteny relationship between apple, strawberry and peach**

| **Duplicated pair** | | **Anchors** | **E value** |
| --- | --- | --- | --- |
| PpCPK3 | FvCPK3 | 249 | 0.00E+00 |
| PpCPK13 | FvCPK13 | 229 | 0.00E+00 |
| PpCPK28 | FvCPK28 | 210 | 0.00E+00 |
| PpCPK6 | FvCPK5 | 164 | 0.00E+00 |
| PpCPK9 | FvCPK9 | 136 | 7.41E-317 |
| PpCPK11 | FvCPK11a | 130 | 0.00E+00 |
| PpCPK21 | FvCPK21 | 107 | 1.33E-267 |
| PpCPK20 | FvCPK20 | 80 | 1.19E-184 |
| PpCPK2 | FvCPK2 | 79 | 1.27E-182 |
| PpCPK9 | MdCPK9a | 77 | 2.40E-213 |
| PpCPK29 | FvCPK29 | 73 | 3.15E-201 |
| MdCPK5b | FvCPK5 | 70 | 7.29E-150 |
| PpCPK2 | MdCPK2 | 68 | 1.24E-191 |
| PpCPK28 | MdCPK28 | 66 | 4.34E-168 |
| PpCPK8b | FvCPK8a | 55 | 5.16E-118 |
| PpCPK2 | FvCPK20 | 49 | 2.28E-103 |
| PpCPK13 | MdCPK13a | 47 | 5.05E-96 |
| PpCPK10 | FvCPK10 | 46 | 5.39E-114 |
| PpCPK20 | FvCPK2 | 46 | 2.90E-102 |
| FvCPK13 | MdCPK13a | 44 | 6.29E-100 |
| PpCPK10 | MdCPK10a | 40 | 1.64E-68 |
| PpCPK9 | MdCPK9b | 39 | 1.32E-79 |
| PpCPK21 | MdCPK21 | 38 | 3.47E-82 |
| FvCPK28 | MdCPK28 | 36 | 3.56E-77 |
| PpCPK29 | MdCPK29 | 32 | 1.17E-85 |
| MdCPK29 | FvCPK29 | 32 | 4.14E-83 |
| MdCPK11 | FvCPK11a | 31 | 1.57E-62 |
| MdCPK20b | FvCPK20 | 28 | 2.58E-53 |
| MdCPK2 | FvCPK2 | 27 | 3.97E-51 |
| MdCPK11 | PpCPK11 | 26 | 1.58E-59 |
| MdCPK2 | FvCPK20 | 24 | 2.17E-41 |
| PpCPK20 | MdCPK20a | 23 | 1.02E-47 |
| PpCPK20 | MdCPK20b | 23 | 4.41E-42 |
| PpCPK6 | MdCPK5b | 19 | 1.03E-46 |
| MdCPK20a | FvCPK20 | 18 | 9.48E-38 |
| PpCPK9 | MdCPK21 | 16 | 2.29E-26 |
| PpCPK8a | FvCPK8b | 15 | 9.20E-24 |
| FvCPK10 | MdCPK10a | 14 | 2.95E-25 |
| PpCPK9 | FvCPK21 | 13 | 3.65E-20 |
| MdCPK26b | PpCPK6 | 11 | 2.91E-25 |
| MdCPK1b | FvCPK1 | 9 | 8.92E-15 |
| MdCPK26b | FvCPK5 | 8 | 1.45E-16 |
| MdCPK13b | FvCPK13 | 8 | 1.75E-11 |
| PpCPK1 | FvCPK1 | 7 | 8.44E-14 |
| MdCPK29 | FvCPK5 | 7 | 1.69E-05 |
| FvCPK9 | MdCPK9a | 7 | 2.46E-15 |
| PpCPK3 | MdCPK3 | 6 | 2.62E-11 |
| PpCPK13 | MdCPK13b | 6 | 2.71E-07 |
| PpCPK4 | MdCPK4b | 6 | 2.88E-08 |
| FvCPK21 | MdCPK9a | 6 | 3.43E-06 |
| FvCPK8a | MdCPK8a | 6 | 1.65E-11 |
| MdCPK17a | PpCPK10 | 5 | 4.86E-06 |
| PpCPK3 | FvCPK29 | 5 | 1.13E-04 |
| PpCPK21 | MdCPK9b | 5 | 6.49E-05 |
| PpCPK8b | MdCPK8a | 5 | 1.29E-09 |
| PpCPK8a | MdCPK32b | 5 | 3.21E-06 |
| MdCPK9b | FvCPK21 | 5 | 1.64E-04 |
| MdCPK32b | FvCPK8b | 5 | 3.99E-08 |
| FvCPK1 | MdCPK1a | 5 | 1.26E-08 |
